# ‘Jus viperinum’: Francis Home (1719–1813) and his experiments on the benefits of viper broth in skin disease

**DOI:** 10.1177/09677720251317804

**Published:** 2025-02-16

**Authors:** Shanghavie Loganathan, Max Cooper

**Affiliations:** 152127 Department of Primary Care and Public Health, 12190 Brighton and Sussex Medical School, Brighton, UK of Great Britain and Northern Ireland

**Keywords:** Viper broth, 18th century, cross-over, dermatology, Edinburgh, snakes

## Abstract

Francis Home (1719–1813) was a Scottish physician and medical author. Here we consider his biography and three brief accounts of experiments on viper (i.e. adder flesh) broth in the treatment of skin disease (‘Herpes or Lepra Gracaeorum’). After a fortnight of treatment one patient was improved but not cured (discontinued due to lack of vipers), one ‘almost cured’ but refused further treatment at ‘disgust’ of discovering its contents, and one dismissed cured. The second case constitutes a basic ‘cross-over’ model as it led to comparison of viper with snail broth in the same patient. Home concluded that viper broth was beneficial for skin disease but his findings did not lead to wider adoption of the treatment. His reported clinical benefits likely arose from improved hydration, protein and cessation of other treatments. Home does not discuss his small sample size or present quantitative outcomes. Home's methods were not influenced by Lind's (1753) methodology for comparing treatments. As both were Edinburgh-trained Scots who served in the British military forces, this reveals the limited communication between clinicians of the day. Home appears to have faced many practical challenges, including accessing vipers, motivating patients’ participation and the risk of concomitant treatment with other drugs.

## Introduction

Snakes have long been used in medicine, most notably in ancient Greece. At that time, non-venomous snakes formed part of healing rituals in the name of Asclepius, the Greek god of medicine. One famous Asclepian temple was on the Greek island of Kos, where in the fifth century BCE Hippocrates (the ‘father’ of Western Medicine) was a pupil. Pilgrims often flocked to such temples in search of healing and here snakes roamed the floors of the room where the sick slept. As well as healing, snakes were more widely associated with wisdom and resurrection. Various explanations have been proposed for why the serpent emerged as a symbol of healing. The foremost explanation lies in the cyclical sloughing (shedding) of snakes’ skin which came to symbolise the rejuvenation associated with medicine.^1^ It may be for this reason too that remedies from snakes have frequently been used in treating skin problems.

Today snakes remain associated with medical practice in various ways. First, it is important to remember that snakebite remains a global health emergency and neglected tropical disease that largely impacts poor and rural populations.^2^ Second, is through modern pharmacological research. This has led to the development of drugs such as captopril, a medication for blood pressure originally isolated from the venom of *Bothrops jararaca* (a South American pit viper).^3^ Third, is through symbolism (especially coats of arms) of medical institutions such as the Royal College of Surgeons of England. The rod of Asclepius remains a commonly adopted symbol of medicine in the form of a snake twisting around a staff.^4^

Snake soup – although not commonly consumed in the English-speaking world – remains a popular Cantonese dish.^5^ It has been touted throughout the centuries for its medicinal benefits. Old Chinese medical books posit medicinal benefits including for bodily ailments, blood and skin quality. Little medical research has considered the health benefits of such treatments.

In Great Britain there are just three indigenous species of snake, only one of which is a viper: the European adder (*Vipera berus*).^6^ This is notable for being the only snake that can live in colder climes, for example in Scotland. Indeed, it is the only snake species commonly found in Scotland. That adders were used in basic medical research in 18th century Scotland is evident in the published studies of Francis Home (1719–1813). These were published in his book ‘Clinical Experiments, Histories, and Dissections’ which was first published in 1780 and appeared in three editions.^7,8^

## Biography of Francis Home (1719–1813)

**Figure 1.**
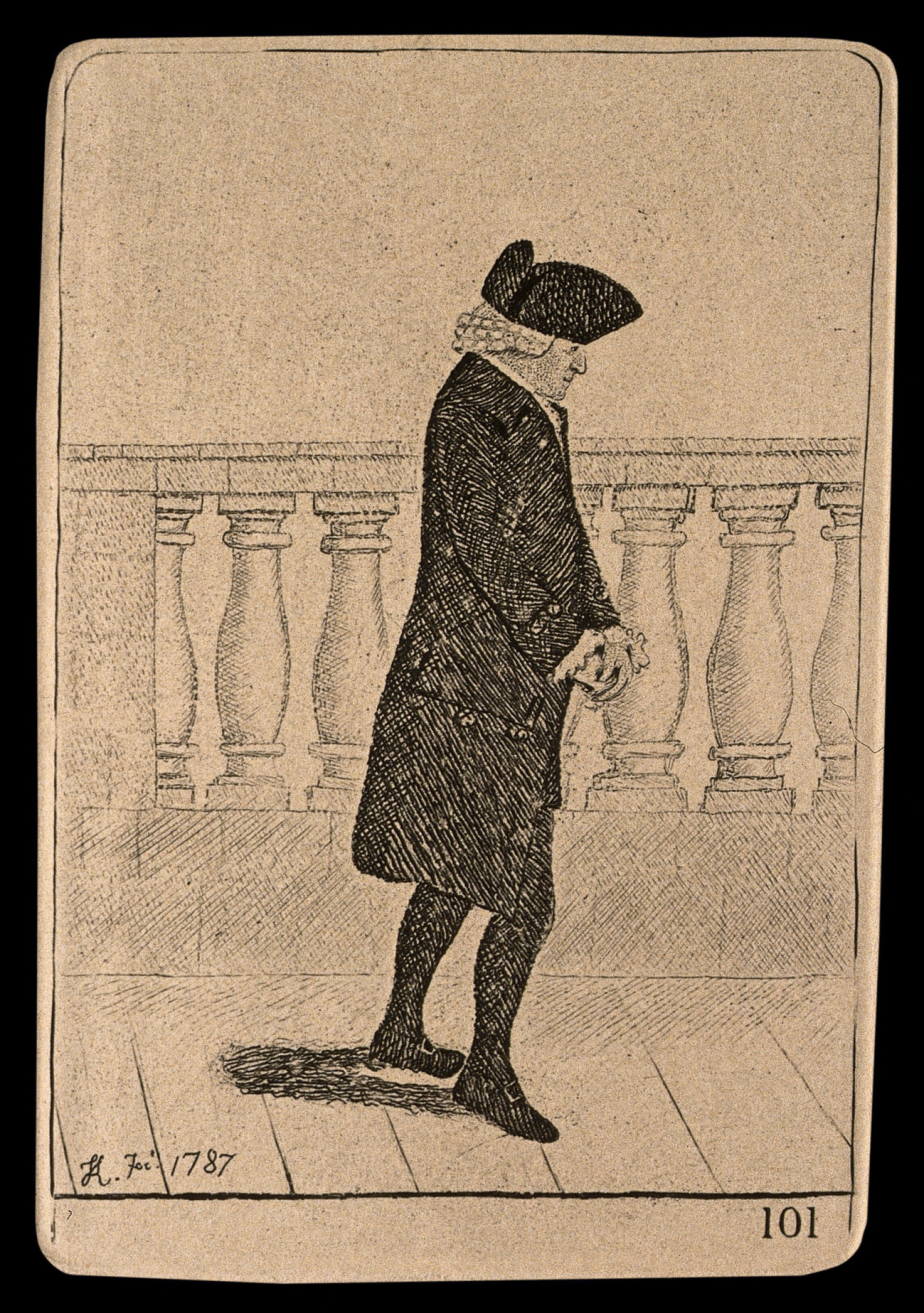
Etching of Francis Home by J, Kay 1787. Image courtesy of the Wellcome Collection.

Francis Home was a Scottish physician notable for his medical books and clinical experiments (Figure 1).^7,8^ He was the son of John Home (an advocate from Bedfordshire, England) and following his education at the Duns Grammar School, completed an apprenticeship under Dr Rattray of Edinburgh.^9^ Subsequently, Home served as regimental surgeon to the Seventh Dragoons during the Austrian War of Succession (1742–1748) and studied at Leiden University during pauses in the campaign.^10,11^ He graduated MD from the University of Edinburgh in 1750. He became a Fellow of the Royal College of Physicians of Edinburgh by 1751.^10^ He then practised as a physician in Edinburgh and is notable for undertaking the first attempt to vaccinate against measles.^12,13^ He was appointed as the first Professor of Materia Medica at the University of Edinburgh in 1768.^14^

Home senior went on to serve as President of the Royal College of Physicians of Edinburgh. He held other roles including serving as President of the Physical Section of the Royal Society of Edinburgh, President of the Harveian Society of Edinburgh and becoming a founding member of the Royal Medical and Select Society.^15^ It is of historical note that one son, James Home (1760–1844), became president of the Royal College of Physicians of Edinburgh (1809–1812) and succeeded his father as Professor of Materia Medica at the university there.^16^ James went on to become Chair of the Practice of Physic at Edinburgh.^16^ A younger one son (also named Francis Home) graduated as a Doctor of Medicine in 1800 and fought in the Peninsular war and at Waterloo.^11^

The contributions of Francis Home (senior) include a gold medal winning essay on experiments on bleaching, a widely used textbook *Principia Medicinae* and the identification of croup (a common childhood respiratory infection) as a distinct clinical condition.^17–19^ Home identified the lack of research surrounding croup and through an analysis of 12 cases identified its predilection for children. The classic clinical signs he noted included voice changes and, through post-mortem examination of children with croup, identified the ‘cavity of the windpipe’ as the affected organ.^19^ Here we discuss Home's three brief experiments on viper broth as a treatment for dermatological conditions.

### ‘Jus viperinum’: An 18th-century recipe for viper broth

In this book, Home does not proffer his own viper broth recipe. It is also unclear who actually captured snakes and prepared the broth for Home. Given the skill and risk involved, it is very likely that these tasks were undertaken by others. An insight into the contents of viper broth can be gleaned from a contemporaneous recipe. Although the recipe book was from Edinburgh it is suggested by the term ‘Lond’ that the recipe originated from the South of England where adders are far more numerous.^20^ It is of note that the recipe mixes viper with chicken meat, perhaps in order to conceal or to temper revulsion at eating snake flesh. The recipe takes a stepwise approach and is detailed below:

Take a middle sized viper, freed from the head, skin and intestines and two pints of water

Boil them to a pint and a half; remove the vessel from the fire and when the liquor is cold let the fat, which congeals upon the surface, be taken off

Into this broth whilst warm put a pullet of a moderate size, drawn and freed from the skin and all the fat but with the flesh intire [sic]

Set the vessel on the fire again that the liquor may boil; then remove it from the fire, take out the chicken and immediately chop its flesh into little pieces

Put these into the liquor again, set it over the fire and as soon as it boils up, pour out the broth after taking off the scum.^20^

### Home's experiments using viper broth to treat dermatological conditions

In his book Home mentions that ancient healers had reported benefits of medication from vipers in the cure of cutaneous diseases. However, he deduced that the evidence was ‘disputed’ with scientists at the time disagreeing over the efficacy of vipers as a medicinal treatment.^8^ Home states that Sir Hans Sloane FRS (1660–1753) used ‘vipers grease’ for ophthalmia (eye disease) and this was likely to be an inspiration for Home's enquiry into the medicinal use of snakes. Home states that Sloane made something of a comparison between two treatments: ‘[Sir Hans] thought that the vipers [sic] grease was material [i.e. important to the treatment process], as, when used alone, it appeared to him to be more successful than oil of olives’. Home reveals his inquisitive mind and desire to test other treatments by stating the following: ‘to ascertain whether this be so or not, would require more experiments than have yet been made. As there is no vipers grease kept in this country [presumably Scotland], we were obliged to make use of hogs lard [instead]’.^8^

**Figure 2.**
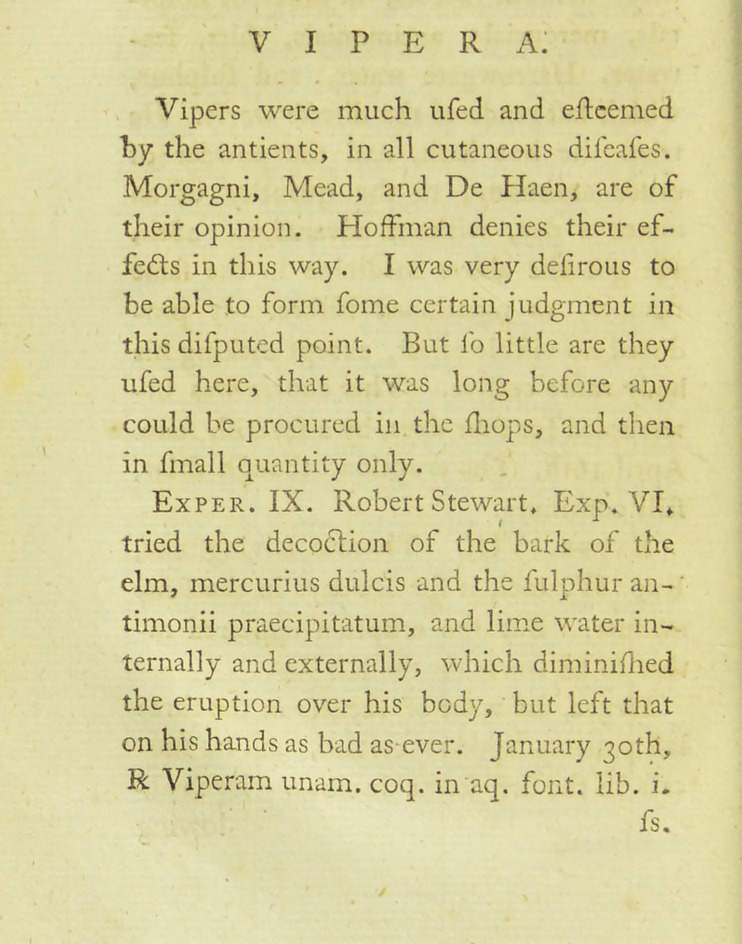
Page 446 from Home's book ‘Clinical Experiments, Histories and Dissection’. Image courtesy of the Wellcome Collection.

**Figure 3.**
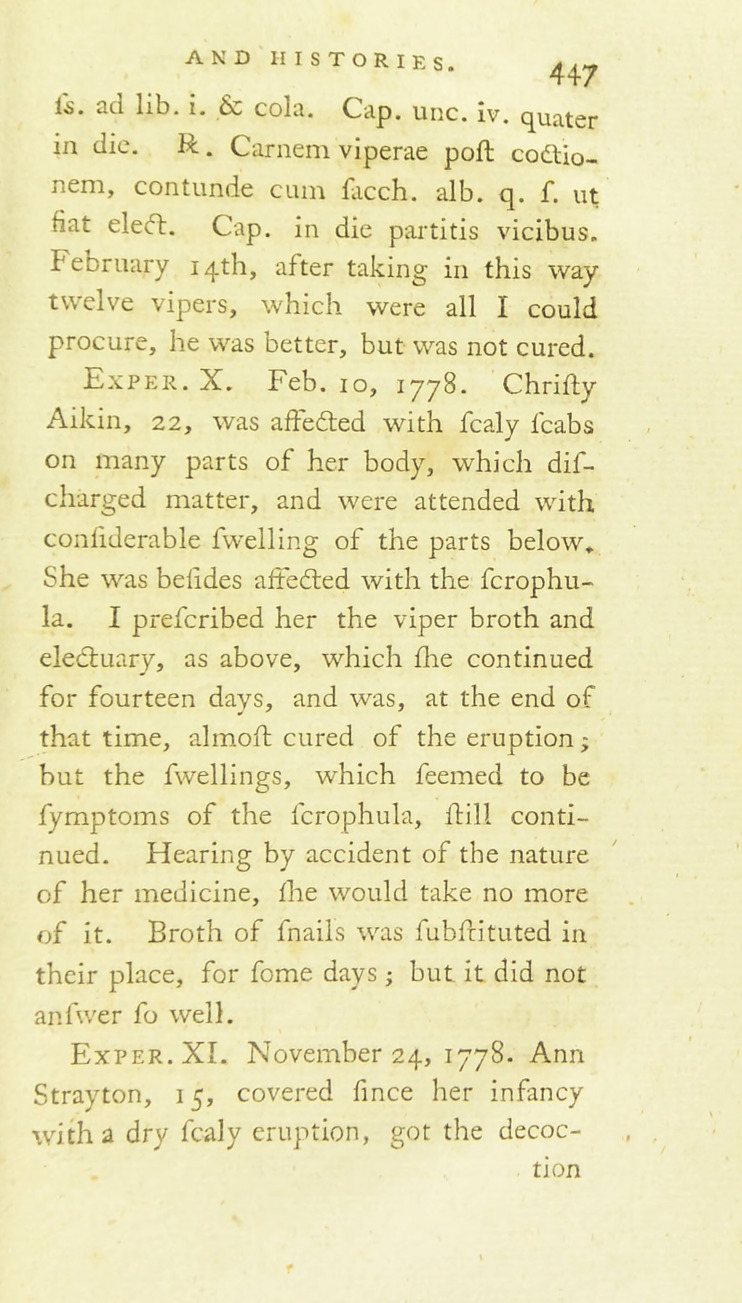
Page 447 from Home's book ‘Clinical Experiments, Histories and Dissection’. Image courtesy of the Wellcome Collection.

**Figure 4.**
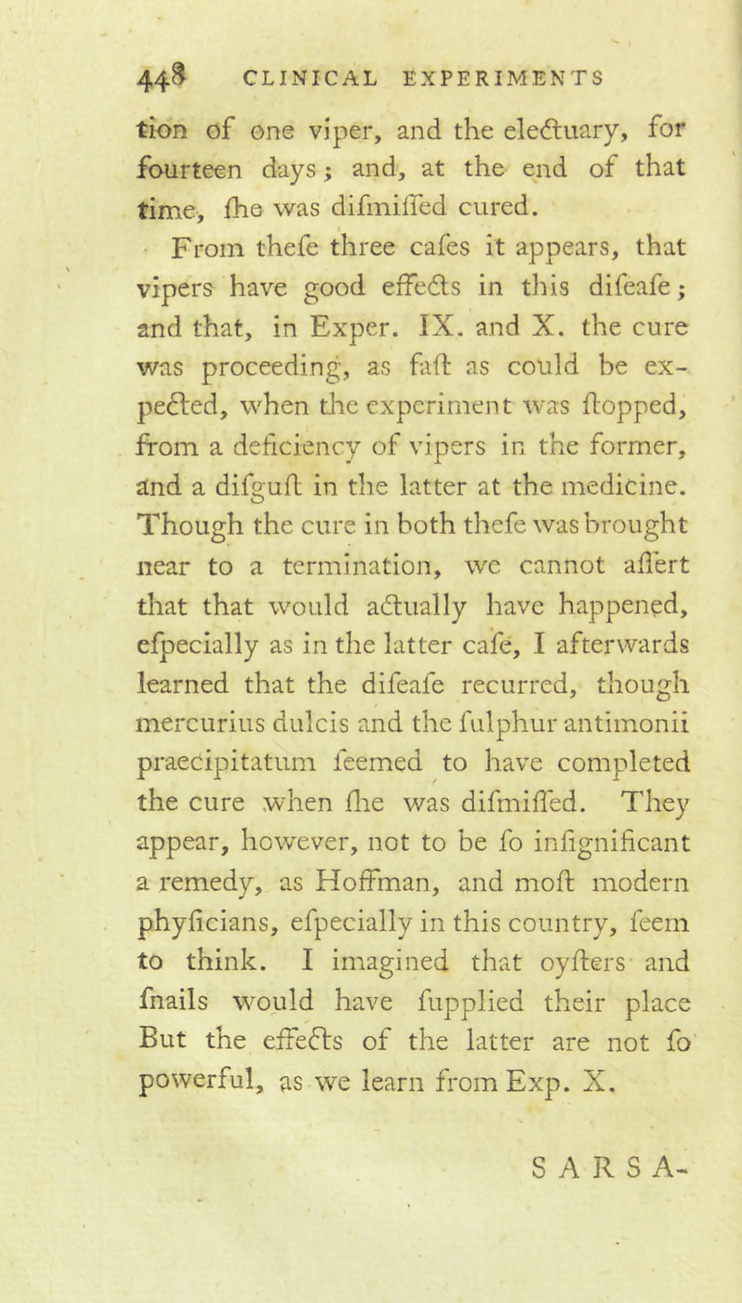
Page 448 from Home's book ‘Clinical Experiments, Histories and Dissection’. Image courtesy of the Wellcome Collection.

Home later describes three separate experiments (i.e. brief case descriptions) to assess the value of ‘viper broth’ as a treatment for dermatological problems in order to ‘form some certain judgment in this disputed point’, (Figures 2–4). Home states that the skin condition he was treating was ‘Herpes or Lepra Gracaeorum’.^8^ He provides his instructions in Latin on the manufacture of the viper broth, including the following (Figure 2): ‘carnem viperae post coctionem contunde cum sacch alb q.f. ut fiat elect’ (After cooking, crush the flesh of the viper with sacch alb [white sugar] q.f. to be elected [i.e., sweetened]).

It is of note that Home added an ‘electuary’, a sweetener (often honey) to improve palatability. While widely used in Home's day, an electuary may have been particularly important with viper broth to disguise its true contents.

Home's experiments took the form of observations of treatment effects in his patients, the first of whom was Robert Stewart (Figure 2). Stewart had previously tried elm bark, mercury, sulphur and limewater which had diminished the ‘eruption’ on his body. No year or age is given in the case history. Home trialled a treatment of viper broth for 14 days, a course which amounted to consuming a total of 12 adders. Stewart's condition did improve but was not cured. Hereafter, Home was unable to procure additional vipers and had to terminate the experiment early. Home attributed the lack of complete cure to the premature termination of viper broth.

The second case was that of 22-year-old Christy Akin (Figures 3 and 4) undertaken in February 1778. She was affected by ‘scrophula’, a condition today considered to be tuberculosis of the lymph glands of the neck. Akin presented with ‘scaly scabs which discharged matter’ and ‘considerable swelling of the parts below’.^8^ She was given viper broth for 14 days and the eruption was ‘almost cured’ but the swelling persisted. The experiment ceased because she realised she had been given viper broth and refused further treatment. She was then switched to snail broth which proved less effective. It is not clear from the account whether Christy knew that she was consuming snails. Home noted that her disease did recur but following the addition of sulphur and mercury she was cured. From Home's account, it appears that she may have taken her custom elsewhere: ‘I afterwards learned that the disease recurred, though mercurius dulcis and the sulphur antomonii praecipitatum seemed to have completed the cure when she was dismissed’.^8^

The final case was that of Ann Strayton, age unknown, who since infancy had suffered with dry, scaly eruptions (Figure 4). She was given viper broth for 14 days in November 1778. On completion, she was ‘dismissed cured’ of the disease.^8^

From these three patients, Home concluded that viper broth produces good effects on cutaneous diseases. He posited that the incomplete cure was due to lack of vipers in the case of Stewart and ‘disgust’ in the case of Akin leading to unfinished treatment.

The stimulus behind Home's brief foray into experimenting with viper broth appears to lie in the works of Sir Hans Sloane. Although Home was personally persuaded of the efficacy of viper broth, there is no evidence to suggest that his findings led to wider acceptance of the treatment in Scotland or elsewhere. Reasons for that likely lay in challenges procuring vipers, revulsion at eating snake flesh and his small sample size. Despite Home's claim that ‘vipers have good effects in this [i.e., skin] disease’, his published results are not persuasive.^8^ That may be because he presents no overarching quantitative or tabular summary to lay bare his findings. For the three cases this amount to: improved but not cured (discontinued due to lack of vipers), one ‘almost cured’ but refused further treatment (at ‘disgust’ of discovering its contents), and one dismissed cured. The lack of a quantitative summary is in contrast to Home's outcomes published in the same book for the treatment of hydrops (fluid retention).^21^

The case of Christy Akin, her revulsion at eating snakes and Home's use of an electuary together suggest that Home's patients did not know what treatment they were taking. This was not unusual in the 18th century, especially for patients who could not read Latin prescriptions. Home does not comment upon the value of patients being blinded to their treatment in reducing bias. It is of historical note that blinding – at least in the form of blindfolding – had already been reported as a method for investigating animal magnetism (Mesmerism) in France in 1784.^22^

Home's second case (Christy Akin) constitutes a basic cross-over (n of one) model, allowing the comparison of two treatments (viper vs snail broth) in the same patient. Home does not, however, repeat this approach of direct comparison in his third case. That a lack of resources (adders) should lead to treatment comparison harks back to the famous natural experiment of Ambroise Pare (1510–1590) that reformed the management of patients after limb amputation on the battlefield.^23^

Home reveals that two weeks were considered a reasonable duration to trial a new medication in patients. Although he uses the term ‘a fair trial’ to describe his experimental approach, he appears to mean that viper broth had been tried for a sufficient duration. Although Home believed his patients were suffering from the same skin diagnosis, it is probable that these were, in fact, multiple. Likely causes include pustular acne and tuberculosis of the neck glands. It is probable that the clinical improvements reported by Home lay in improved hydration, intake of protein and cessation of more toxic medications that were widely used at this time. Even so, it is possible that his patients may have continued to ‘mix’ treatments unbeknown to Home.

Home's attempts at using viper broth constitute empirical (i.e. first-hand observation) rather than higher-order comparative enquiry. This was typical of most medical experiments undertaken by doctors of the day. Home's approach to experimentation did not emulate Royal Naval Surgeon and fellow Scot James Lind (1716–1794) who undertook the first controlled comparison of treatments in 1747.^24^ This is surprising given that other British military doctors subsequently published the results of controlled trials in the 18th century, most notably the surgeons Vage in 1759 and Thomas Trotter (1760–1832) in 1792.^25,26^ The reasons for this are unclear but may be because Home was resident in Edinburgh and his army service brief. Lind, by contrast, served in the Royal Navy for decades and lived near Portsmouth on the South coast of England.

## Conclusion

Francis Home was a notable 18th-century Scottish physician who published medical texts and undertook observational clinical experiments. His brief experiments on the use of viper broth to treat dermatological conditions were undertaken at a critical period in the development of methodologies for assessing the efficacy of medical treatments.^23^ The practical circumstances under which Home conducted his work made formal comparisons of single treatments challenging to implement. A further issue is that Home's patients may have been likely to report positive outcomes given the esteem in which physicians were held. It may also be that Home's favourable judgement on the value of viper broth was influenced by additional information such as unpublished accounts from clinical colleagues. In contrast to his positive conclusion about the benefits of viper broth Home makes the following insightful observation about the danger of making assumptions about clinical outcomes in such cases: ‘Though the cure in both these [i.e., his first two cases] were brought near to a termination, we cannot assert that would actually have happened’.^8^

Home's methods suggest that he was not influenced by Lind's controlled study comparing different treatments for scurvy. This may appear surprising given that both were Edinburgh-trained Scots who served in the British military forces. Nevertheless, it underlines the limited communication between clinicians of the day. Home's foray into studying viper broth reveals an inquisitive mind but his small sample size highlights the difficulty of reaching a definitive conclusion about treatment efficacy in the 18th century. Home faced many practical challenges such as accessing vipers, motivating patients’ participation and preventing concomitant treatment with other products. These circumstances ultimately offered insufficient evidence to present a persuasive conclusion about the benefit of viper broth in skin disease. Despite that, Francis Home’s place in the history of medicine is secure and lies in his enquiring mind, other experiments and many practical contributions. These include his description of croup, attempt at vaccinating against measles and multiple medical texts.
